# Modulation of dopaminergic neurotransmission in rat striatum upon *in vitro* and *in vivo* diclofenac treatment^1^

**DOI:** 10.1111/j.1471-4159.2007.05141.x

**Published:** 2008-04-01

**Authors:** Elisaveta Milusheva, Mária Baranyi, Agnes Kittel, Adam Fekete, Tibor Zelles, E Sylvester Vizi, Beáta Sperlágh

**Affiliations:** * Institute of Neurobiology (former Institute of Physiology), Bulgarian Academy of Sciences Sofia, Bulgaria; † Institute of Experimental Medicine, Hungarian Academy of Sciences Budapest, Hungary

**Keywords:** diclofenac, dopamine release, H_2_O_2_, oxidative stress, striatum

## Abstract

Diclofenac (DCF) is a widely used non-steroidal anti-inflammatory drug, which also act as a mitochondrial toxin. As it is known that selective mitochondrial complex I inhibition combined with mild oxidative stress causes striatal dopaminergic dysfunction, we tested whether DCF also compromise dopaminergic function in the striatum. [^3^H]Dopamine ([^3^H]DA) release was measured from rat striatal slices after *in vitro* (2 h, 10–25 μmol/L) or *in vivo* (3 mg/kg i.v. for 28 days) DCF treatment. *In vitro* treatment significantly decreased [^3^H]DA uptake and dopamine (DA) content of the slices. H_2_O_2_ (0.1 mmol/L)-evoked DA release was enhanced. Intracellular reactive oxygen species production was not significantly changed in the presence of DCF. After *in vivo* DCF treatment no apparent decrease in striatal DA content was observed and the uptake of [^3^H]DA into slices was increased. The intensity of tyrosine hydroxylase immunoreactivity in the striatum was highly variable, and both decrease and increase were observed in individual rats. The H_2_O_2_-evoked [^3^H]DA release was significantly decreased and the effluent contained a significant amount of [^3^H]octopamine, [^3^H]tyramine, and [^3^H]β-phenylethylamine. The ATP content and adenylate energy charge were decreased. In conclusion, whereas *in vitro* DCF pre-treatment resembles the effect of the mitochondrial toxin rotenone, *in vivo* it rather counteracts than aggravates dopaminergic dysfunction.

*J. Neurochem.* (2008) **105,** 360–368.

Diclofenac (DCF), a widely prescribed non-steroidal anti-inflammatory drug (NSAID), causes adverse effects in liver and gastrointestinal tract. Although a great number of studies were devoted to clarify the underlying mechanisms, they are still not clear. *In vitro* experiments have shown that DCF affected mitochondrial respiration, inhibited ATP synthesis, and collapsed mitochondrial membrane potential (Petrescu and Tarba 1997; Moreno-Sanchez *et al.* 1999). Further, it was found that DCF causes apoptosis in hepatocytes, most likely via an oxidative stress-induced opening of mitochondrial permeability transition pore (Gomez-Lechon *et al.* 2003), and according to Inoue *et al.* (2004) the molecular mechanism of DCF-induced apoptosis involves as an initial step the generation of reactive oxygen species (ROS).

Compromised ATP biosynthesis caused by DCF was also observed in kidney mitochondria and rat thymocytes (Krause *et al.* 2003; Ng *et al.* 2006). Although it is known that brain cells are highly dependent on energy production, which may be compromised by inhibition of mitochondrial oxidative phosphorylation, so far a limited number of studies are known concerning DCF effects in brain. Decreased dopamine (DA) levels have been demonstrated in mice brain after *in vivo* DCF treatment (Hassanein *et al.* 2004). Recently, two other NSAIDs, tolmetin and sulindac, were shown to reduce significantly DA levels in rat striatum (Dairam *et al.* 2006).

In our recent study, we showed that *in vitro* and *in vivo* treatment with the mitochondrial complex I inhibitor rotenone decreased DA levels in striatum, inhibited DA uptake, and enhanced the sensitivity of striatal DA release to oxidative stress induced by H_2_O_2_ (Milusheva *et al.* 2005), thus reproducing the neurochemical features of Parkinson's disease. We also demonstrated that rotenone and H_2_O_2_ have a supra-additive impact on the release of monoamine transmitters and that even a minimal mitochondrial deficit could predispose neurons to the harmful effect of subsequent oxidative stress (Milusheva *et al.* 2003).

As DCF was recognized as a mitochondrial inhibitor, too, the question arises whether it may mimic the effects of rotenone on striatal dopaminergic neurotransmission. Therefore, we studied the effect of acute and chronic DCF treatment on the release of DA from rat striatal slices under conditions of mild oxidative challenge.

## Materials and methods

### Chronic diclofenac treatment: implantation of osmotic pumps and animal care

All animal experiments were performed in accordance with the National Institute of Health Guide for the Care and Use of Laboratory Animals and were approved by the Institutional Animal Care and Use Committee of the Hungarian Academy of Sciences.

We used male Sprague–Dawley rats (280–320 g). Alzet osmotic minipumps (Alzet Corporation, Palo Alto, CA, USA) were filled with DCF dissolved in equal volumes of dimethylsulfoxide (DMSO) and polyethylene glycol (PEG-300) or with the solvent alone. Pumps were kept in sterile 0.9% saline at 37°C overnight before the operation. Ketamine (75 mg/kg) and xylazine (10 mg/kg) were injected intramuscularly as anesthetics. Pumps were implanted under the skin on the back of each animal and were attached to the right jugular vein by a catheter. Sham-operated control rats received DMSO : PEG (1 : 1) only. The treated rats received 3 mg/kg/day DCF (calculation based on body weight at the time of surgery) for 28 days. Our preliminary experiments showed that pre-treatment with higher dose of DCF (5–6 mg/kg/day) elicited toxic effects, with high mortality of the experimental animals. Following surgery, rats were monitored for behavior, weight, and overall health.

### [^3^H]Dopamine release

Rats were decapitated after a short ether stress and the brain was quickly removed. Then the striatum was dissected out and sliced into 400-μm thick sections with a McIlwain tissue chopper (Bachofer, Reutlingen, Germany). In some experiments, a full cross-section of the brain, including the striatum and cortex, was cut for immunohistochemistry. All manipulations were performed on ice.

Slices were incubated in 1 mL of Krebs solution containing 5 μCi [^3^H]dopamine ([^3^H]DA) for 45 min and were continuously gassed with a mixture of 95% O_2_ and 5% CO_2_ at 37ºC. After incubation the slices were rinsed and transferred to tissue chambers and perfused continuously with modified Krebs solution at a rate of 0.5 mL/min. After a 60-min pre-perfusion 3-min samples were collected and assayed for [^3^H]DA. The samples for HPLC analysis of [^3^H] metabolites were acidified by 15 μL of 1.9 mol/L perchloric acid during the collection.

Oxidative stress was mimicked by addition of H_2_O_2_ to the Krebs solution starting at the ninth minute of collection. In a separate set of experiments striatal slices from non-treated rats were kept for 2 h in Krebs solution containing 10 or 25 μmol/L DCF, then rinsed with normal Krebs solution and loaded with the isotope.

At the end of the experiment, slices were homogenized in 0.5 mL of 10% trichloroacetic acid. A 0.5-mL aliquot of the superfusate and 0.1 mL of the tissue supernatant were added to 2 mL of scintillation cocktail (Ultima Gold; Packard, Canberra, Australia). Tritium was measured with a Packard 1900 TR liquid scintillation counter using an internal standard. The release of tritium was calculated in Bq/g and expressed as the percentage of the amount of radioactivity in the tissue at the sample collection time (fractional release, %). The net release evoked by H_2_O_2_ was calculated by the area-under-the-curve method, i.e. subtracting the resting release calculated from the pre-stimulation period, from the release measured during H_2_O_2_. The tissue tritium uptake was determined as the sum release + the tissue content after the experiment and expressed in Bq/g, which reflects the content of radioactivity of the slices after the 60 min washout period, i.e. the radioactivity that is specifically taken up by the tissue.

### HPLC determination of dopamine content, [^3^H] metabolites, and adenine nucleotides

For the simultaneous measurement of biogenic amines and adenine nucleotides a liquid–liquid two-dimensional reversed-phase and ion pair reversed-phase chromatographic separation was developed (Baranyi *et al.*, 2006). Briefly, a Gilson liquid chromatographic System with 715-operation software (Gilson Medical Electronics Inc., Middletown, WI, USA) was used. For the enrichment a ‘trap-column’ [15–25 μm Nucleosil C-18 (20 × 4.0)] (Sigma, St. Louis, MA, USA) was inserted into a loop position. The separation of neurotransmitters was performed on a 3 μm Discovery C18 HS F5 (150 × 4.0 mm) (Sigma) analytical column. The analysis of adenine nucleotides was carried out at the first 10 min with reversed-phase buffer. The flow rate increased from 0.6 to 1.0 mL/min, linearly. The separation of catecholamines was accomplished with ion pair reversed-phase buffer at constant flow rate 1.0 mL/min. The effluents were monitored by UV, electrochemical, and radiochemical detector. They were connected in a cascade line. The identification of the tritium labeled compounds was based upon the known retention times of unlabeled standards.

At the end of release experiments the tissue slices were immediately frozen in liquid nitrogen. The perfusion fluid was centrifuged at 300 *g* (4500 rpm) for 10 min at 0–4°C; the supernatant was kept at −20°C until analysis. For the analysis, 545-μL sample volumes were diluted with 5 μL of 10^−5^ mol/L dihydroxybenzyl amine as an internal standard and 500 μL were injected.

After the preparation of striatum of chronically treated rats the native tissue was frozen by liquid nitrogen. In case of *in vitro* experiments, the slices were frozen after DCF pre-treatment. The control slices were kept in normal Krebs solution before freezing. The weighed frozen tissue was homogenized in an appropriate volume of ice-cold 0.1 mol/L perchloric acid that contained theophylline (as an internal standard) at 10 nmol/mL concentration and 0.5 mmol/L sodium metabisulfite (antioxidant for biogenic amines). The suspension was centrifuged at 300 *g* (4500 rpm) for 10 min at 0–4°C. The perchloric anion was precipitated by addition of 10 μL of 1 mol/L KOH to 190 μL of the supernatant. The precipitate was then removed by centrifugation at 3509 *g*. The supernatant was kept at −20°C until analysis. The pellet was saved for protein measurement according to Lowry *et al.* (1951). Tissue content of DA, 3,4-dihydroxyphenylacetic acid (DOPAC), and homovanilic acid (HVA) were expressed in pmol/mg protein.

### Intracellular ROS measurement

After decapitation coronal sections (300 μm) of the striatum were cut with a vibratome (Vibratome 1000; TPI, St. Louis, MO, USA), and transferred into a mesh-bottom holding chamber containing artificial CSF (in mmol/L: NaCl 126, KCl 2.5, NaHCO_3_ 26, CaCl_2_ 2, MgCl_2_ 2, NaH_2_PO_4_ 1.25, and glucose 10) bubbled with a mixture of 95% O_2_ + 5% CO_2_, leaving the final pH at 7.4. After 25-min incubation at 32.5°C, the slices were kept at 25°C until the onset of the experiments. ROS production was monitored by using 5-(and-6)-chloromethyl-2′,7′-dichlorodihydrofluorescein diacetate (CM-H_2_DCFDA) sensitive to peroxynitrite, hydrogen peroxide, and hydroxyl radical. Slices were loaded with 45 μmol/L CM-H_2_DCFDA for 45 min at 25°C (Fekete *et al.* 2007). Then they were submerged and superfused (3.5 mL/min) in an experimental chamber mounted on a Gibraltar BX1 platform (Burleigh, Fishers, NY, USA) and viewed with a 10× water immersion objective (Olympus, Hamburg, Germany) on an Olympus BX50WI upright microscope. Slices were excited at 488 ± 5 nm with a T.I.L.L. Polychrome II monochromator (Planegg, Germany). The emitted light (535 ± 25 nm) was detected by a cooled CCD camera (Photometrics Quantix, Photometrix, Tucson, AZ, USA) and the system was controlled with the Axon Imaging Workbench 4.0 software (Axon Instruments, Molecular Devices, Union City, CA, USA). Visualization was performed by differential interference contrast imaging. Intensity changes were measured off-line. Following the subtraction of the autofluorescence of an unloaded slice the actual intensity values were normalized to an exponential curve fitted to the baseline. Experiments on loaded slices but without drug treatment confirmed the exponential nature of the baseline.

Level of intracellular superoxide was measured by the superoxide-selective dye hydroethidine (HEt). The slices were bulk loaded with 60 μmol/L HEt for 25 min at 25°C. Loading solutions were prepared freshly for each experiment. We excited HEt at 495 ± 5 nm and detected emitted light (605 ± 37.5 nm) by the cooled CCD imaging system described above.

### Immunohistochemistry

Non-treated rats, sham-operated controls, and DCF chronically treated animals were decapitated after a short ether stress and the brain was quickly removed. The upper part of the striatum with adjacent cortex was immersed in 4%*p*-formaldehyde, 0.5% glutaraldehyde, and 15% saturated picric acid in 0.1 mol/L phosphate buffer, pH 7.4, for 6 h. The tyrosine hydroxylase (TH)-staining was performed as described before (Milusheva *et al.* 2005). Avidin biotinylated complex reaction and detection of antigen–antibody complexes was performed according to the Vector Laboratories (Burlingame, CA, USA) prescription. Stained sections were washed in water, dehydrated in xylene, and mounted in Canada balsam. Staining was investigated with an Olympus CH30 microscope at 10–40 magnifications. Pictures were taken by Olympus digital camera.

### Materials

The following materials were used: [^3^H]DA (specific activity 39 Ci/mmol) from Amersham (Little Chalfont, UK); H_2_O_2_ (Reanal, Budapest, Hungary); DCF, rotenone, DMSO, and PEG-300 (Sigma, St Louis, MO, USA); and CM-H_2_DCFDA (Molecular Probes, Eugene, OR, USA).

The composition of the Krebs solution was the following: (in mmol/L) NaCl 113, KCl 4.7, CaCl_2_ 2.5, KH_2_PO_4_ 1.2, MgSO_4_ 1.2, NaHCO_3_ 25, Na_2_EDTA 0.03, ascorbic acid 0.3, and glucose 11.5. All solutions were prepared on the day of use.

### Data analysis

All data were expressed as means ± SEM of *n* observations. The statistical analysis was made by anova followed by the Bonferroni test (multiple comparisons) or Student's *t*-test (pair-wise comparisons). Values of *p* < 0.05 were considered statistically significant.

Adenylate energy charge (EC), introduced by Atkinson (1968), was calculated accordingly:

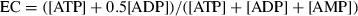


The energy charge (EC) value represents the energy resources of a cell as a function of the concentration of nucleotides. A biological system is fully charged when ATP dominates over other adenine nucleotides and the corresponding EC is close to one.

## Results

### *In vitro* diclofenac treatment

**Fig. 1:**
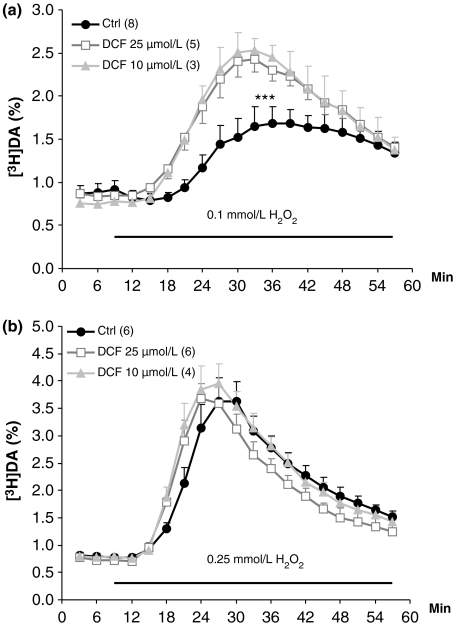
Acute DCF pre-treatment of striatal slices (2 h at 37°C, continuous oxygenation) before incubation with the isotope increases the sensitivity of dopaminergic neurotransmission to oxidative stress. H_2_O_2_ was added to the perfusion fluid at the third fraction in (a) 0.1 mmol/L and (b) 0.25 mmol/L concentration. Control, filled circles; DCF 10 μmol/L, filled triangles; DCF 25 μmol/L, open squares. [^3^H]DA release is expressed as fractional release (for calculation see Materials and methods), as a function of time. Means ± SE of *n* observations are presented. The number of experiments is indicated in the legend. ***p < 0.001, significance versus the control non-pre-treated slice.

**Fig. 2:**
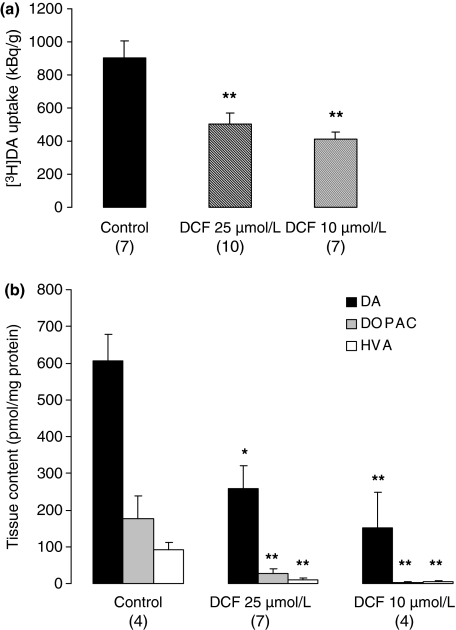
Pre-treatment of striatal slices by DCF decreases [^3^H]DA uptake (a) and dopamine content (b). The concentration of DCF is indicated at the bottom of respective columns. The results are expressed in kBq/g [^3^H]DA uptake and pmol/mg protein (dopamine content), respectively. Means ± SE of *n* observations are presented. The number of experiments is indicated below the columns.*p < 0.05, **p < 0.01, significance versus the control non-pre-treated slice.

In control experiments [^3^H]DA release was measured under basal conditions and in response to H_2_O_2_. After 60-min pre-perfusion, the basal efflux measured in the first 3-min sample was 0.84 ± 0.046% (*n* = 11), which remained relatively constant during the subsequent sample collection. H_2_O_2_ (0.1–0.25 mmol/L) itself elicited a dose-dependent elevation of [^3^H]DA release (Fig. 1a and b). This effect of oxidative stress was significantly enhanced in DCF pre-treated slices, but only at the lower concentration of H_2_O_2_ (0.1 mmol/L) applied (Fig. 1a). It should be pointed out, however, that the amounts of radioactivity released in response to 0.1 mmol/L H_2_O_2_ were similar in slices pre-treated by 10 or 25 μmol/L DCF (14.76 ± 1.65%, *n* = 3, vs. 14.86 ± 1.14, *n* = 5; *p* > 0.05), respectively. Most likely 10 μmol/L of DCF was already enough to cause maximum sensibility of striatal slices to a mild oxidative stimuli. Similarly, [^3^H]DA uptake and DA content of pre-treated slices were decreased, independently of the concentration of DCF (Fig 2a and b). The content of DOPAC and HVA was also lower in pre-treated slices, suggesting a compromised synthesis and decreased turnover of DA.

**Fig. 3:**
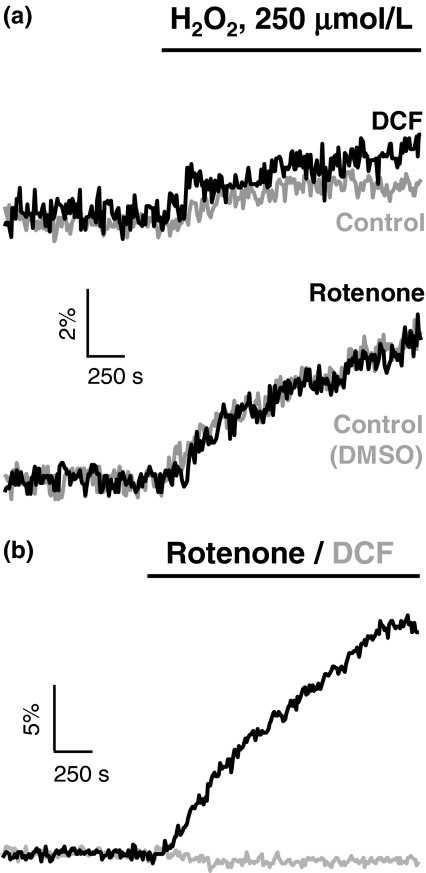
*In vitro* measurement of ROS and superoxide level on striatal slices induced by DCF and rotenone. (a) DCF (25 μmol/L, black, upper trace; *n* = 2) or rotenone (5 μmol/L, black, lower trace; *n* = 3) did not potentiate intracellular ROS production evoked by H_2_O_2_ (0.25 mmol/L) compared with vehicle treatment (water and DMSO, respectively; grey traces; *n* = 3). Treatment by DCF or rotenone for 1 h preceded H_2_O_2_ application. ROS level was monitored by using CM-H_2_DCFDA. Normalized ROS curves are presented for each treatment. (b) Rotenone (5 μmol/L; black line; *n* = 3) induced an increase in superoxide level while DCF (25 μmol/L; grey line; *n* = 3) did not as measured by HEt.

In order to check how DCF influence the intracellular level of ROS, we used the dye CM-H_2_DCFDA sensitive to H_2_O_2_ (Fekete *et al.* 2007). Perfusion of 0.25 mmol/L H_2_O_2_ elevated the level of ROS in striatal slices kept under similar conditions than in release experiments. Pre-treatment of the slices with 25 μmol/L DCF influenced neither the baseline level of ROS (data not shown) nor the fluorescence increase evoked by the administration of H_2_O_2_ (Fig. 3a, upper trace). Similarly, pre-treatment with the mitochondrial complex I inhibitor rotenone (5 μmol/L) failed to affect both the baseline (data not shown) and the 0.25 mmol/L H_2_O_2_-evoked increase of ROS level when compared with slices pre-treated only by DMSO (the solvent of rotenone) (Fig. 3a, lower trace). As measured by the superoxide-selective dye HEt, administration of 25 μmol/L DCF did not have any effect on superoxide level unlike 5 μmol/L of rotenone, which increased it (Fig 3b).

### *In vivo* DCF i.v. application

**Fig. 4:**
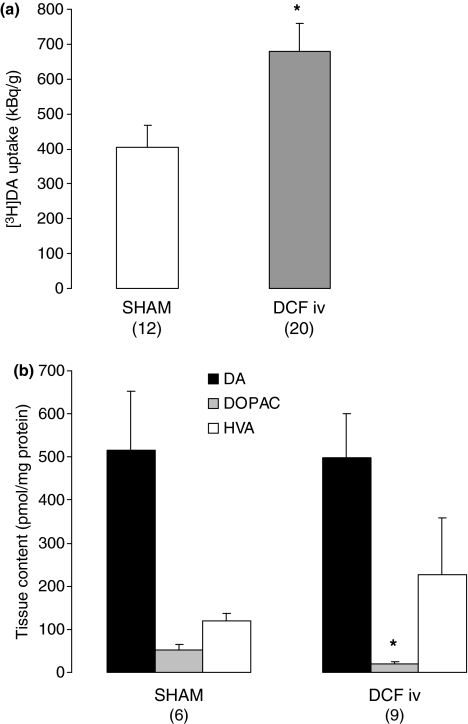
[^3^H]DA uptake (a) increased while dopamine content (b) remained unchanged in striatal slices from rats that have been chronically treated with DCF (3 mg/kg/day) for 28 days. Note that DOPAC was decreased. Sham-operated control rats received DMSO : PEG (1 : 1) only. Continuous infusion of the drug was achieved by subcutaneous implanted Alzet mini-pump connected to the right jugular vein via catheter. The results are expressed in Bq/g [^3^H]DA uptake and pmol/mg protein (dopamine content), respectively. Means ± SE of *n* observations are presented. The number of experiments is given in parentheses.*p < 0.05, significance versus the sham-operated control.

**Fig. 5:**
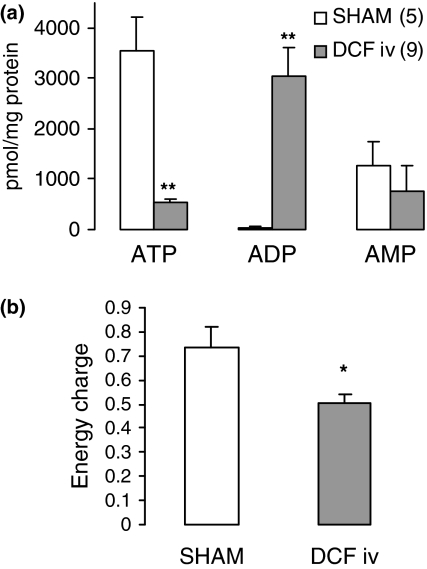
Chronic DCF treatment decreases ATP content (a) and the energy charge (b) of striatal slices. Sham controls, open bars; DCF i.v. treated animals, filled bars. The number of experiments is given in parentheses. Means ± SE of *n* observations are presented.*p < 0.05, **p < 0.01, significance versus the sham-operated control.

In contrast to the previous set, the uptake of tritium was enhanced in slices of chronically treated rats (Fig. 4a). However, there was no difference between the endogenous DA content of sham-operated controls and of chronically treated rats. The amount of DOPAC, on the other hand, was significantly decreased, while HVA showed a tendency toward increase (Fig. 4b). Nucleotides composition was significantly changed in the slices from chronically treated rats (Fig. 5a). A decrease in ATP content, with a simultaneous increase in ADP content could be observed, resulting in the reduction of EC when compared with the sham-operated controls (Fig 5b).

**Fig. 6:**
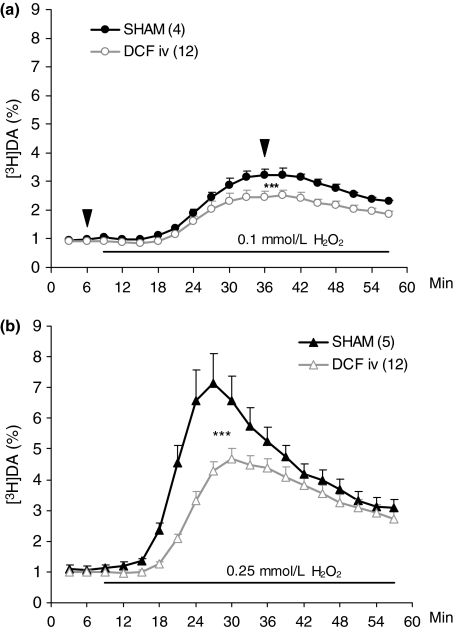
Chronic i.v. DCF treatment decreases the sensitivity of striatal dopaminergic neurotransmission to oxidative stress. (a) H_2_O_2_ 0.1 mmol/L was added to the perfusion fluid at the third fraction; (b) H_2_O_2_ 0.25 mmol/L. Open symbols, sham-operated controls; filled symbols, DCF i.v. treated rats. Means ± SE of *n* observations are presented. The numbers of experiments are given in parentheses. ***p < 0.001, significance versus the sham-operated controls. Black downward triangles indicate the samples which were analyzed for tritium distribution.

In contrast to stimulation of H_2_O_2_-evoked [^3^H]DA release by acute DCF treatment, in the chronically DCF-treated animals, we observed a decreased response to oxidative stress (Fig. 6a). The increase of [^3^H]DA outflow after the onset of oxidative condition developed with some delay and did not reach the level of sham-operated control at the higher concentration of H_2_O_2_ applied (Fig. 6b).

**Fig. 7:**
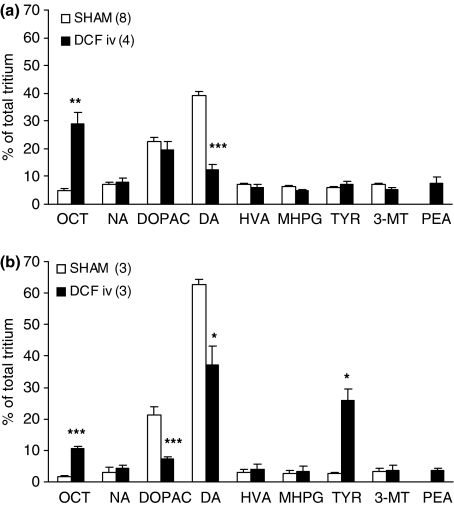
Chronic i.v. DCF treatment decreases the proportion of tritiated DA and 3,4-dihydroxyphenylacetic acid (DOPAC) in the effluent, however it increases the proportion of trace amines: octopamine (OCT), tyramine (TYR), and β-phenylethylamine (PEA). Tritium content of the samples indicated by arrows on Fig 6a was analyzed by HPLC. The samples contained tritiated DA, DOPAC, homovanilic acid (HVA), 3-methyl-4-hydroxyphenyl glycol (MHPG), 3-methyltyramine (3-MT), noradrenaline (NA), OCT, TYR, and PEA. (a) Resting effluent, collected 6 min after the start of collection period. (b) Effluent collected during the peak of the effect of 0.1 mmol/L H_2_O_2_ at 36 min. Means ± SE of *n* observations are presented. The numbers of experiments are given in the parentheses. *p < 0.05, **p < 0.01, ***p < 0.001, significance versus the sham-operated controls.

HPLC analysis of the composition of tritium label in the effluent was performed in two fractions (indicated by black downward triangles at Fig. 6a): under resting conditions and in response to H_2_O_2_ application for 27 min (corresponding to the maximum release). Under resting conditions the effluent contained tritiated DA, DOPAC, HVA, 3-methyl-4-hydroxyphenyl glycol, 3-methyltyramine, noradrenaline, octopamine (OCT), and tyramine (TYR) (Fig. 7a). In the fraction corresponding to the maximum effect of 0.1 mmol/L H_2_O_2_ treatment, the proportion of DA was higher as a percentage of the whole tritium label (62.69 ± 1.44%, *n* = 3, *p* < 0.001 vs. resting: 38.98 ± 1.78%, *n* = 8) (Fig. 7a and b). In DCF treated rats (black bars) significant changes in the composition of tritium label were observed in the presence of H_2_O_2_. The proportion of DOPAC and DA decreased, whereas that of OCT and TYR significantly increased (Fig 7b). Decrease in DA and increase in OCT was also observed in the sample before H_2_O_2_ application i.e. under resting conditions (Fig 7a). In addition, another trace amine (TA), β-phenylethylamine (PEA) also appeared in the effluent both under resting conditions and under the treatment of H_2_O_2_. Similar changes were observed with the higher concentration of H_2_O_2_ (0.25 mmol/L, data not shown). It should be noted that we could not observe the appearance of DA quinone, the toxic oxidative metabolite of DA, detected in our previous study in rats chronically pre-treated with rotenone and exposed to H_2_O_2_ (Milusheva *et al.* 2005).

**Fig. 8:**
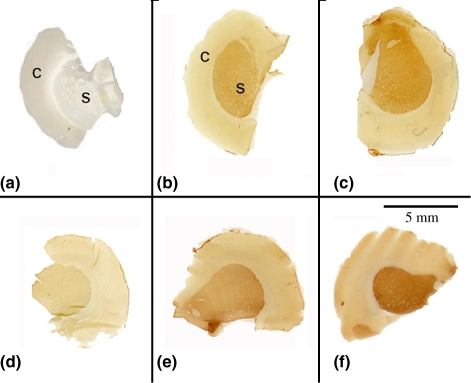
Immunostaining for TH in the striatal brain sections of Sprague–Dawley rats. Staining was performed on 40-μm Vibratome sections of the brain tissue blocks containing the upper part of the striatum with adjacent cortex. Fixation was performed by 4%*p*-formaldehyde, 0.5% glutaraldehyde, and 15% saturated picric acid in 0.1 mol/L phosphate buffer, pH 7.4. Dilution of applied TH antibody (Sigma) was 1 : 1000. Detection of antigen–antibody complexes was performed by DAB as chromogen. Original magnification: 10× S, striatum; C, cortex. (a) Control staining. TH-antibody was omitted from the incubation medium. Brain section from untreated animal. (b) Immunostaining for TH in the brain section of untreated animal. Strong immunoreactivity in the striatum. (c) Striatal brain section of a sham-operated Sprague–Dawley rat. Intensity of immunostaining for TH antibody is comparable with the control staining. (d, e, and f) Different intensity of the TH-staining in the striatal sections after long-term (4 weeks) of DCF treatment (selected samples from the results of TH-immunostaining of eight treated animals). (d) Significantly weaker immunoreactivity in the striatum (two cases). (e) Intensity of the TH-staining is comparable with the staining found in the striatal sections of sham-operated or control animals (two cases). (f) TH-immunoreactivity is stronger after DCF treatment than control (four cases).

Whereas no difference was observed in TH-immunoreactivity in the striatum between sham-operated and non-treated rats (Fig. 8, upper trace), the staining intensity was very variable in different rats undergoing chronic i.v. DCF (3 mg/kg/day) infusion. Some of these rats showed intense TH-immunoreactivity, while low TH-staining was found in about 28% of the chronically treated animals, indicating the loss of DA-synthesizing capacity of dopaminergic nerve terminals (Fig. 8, lower trace). These observations are consistent with poor penetration of the blood–brain barrier by DCF, reported by Dehouck *et al.* (1992), and reflect the individual variations among experimental animals.

## Discussion

Our *in vitro* results showed a significant decrease of both DA content and [^3^H]DA uptake and an enhancement of the release of [^3^H]DA induced by low H_2_O_2_ concentration (0.1 mmol/L) in response to acute DCF treatment, similarly to that observed in our previous study in slices, undergone *in vitro* rotenone treatment (Milusheva *et al.* 2005).

When we measured the changes in ROS level under conditions of superimposed H_2_O_2_ stimulation, DCF failed to enhance the effect of 0.25 mmol/L H_2_O_2_ both in the ROS-related fluorescence assay and in the release experiments. Similarly, 25 μmol/L DCF did not influence superoxide level contrary to rotenone, which induced an immediate superoxide production after its administration. This is most likely because of the double face action of DCF on free radical production, i.e. it could also act as a free radical scavenging antioxidant (Sairam *et al.* 2003; Tang and Liu 2006). The capability of DCF to induce mitochondrial dysfunction, e.g. impaired ATP synthesis (Bort *et al.* 1998) does not necessarily results in the increase of superoxide or other ROS levels. Although Gomez-Lechon *et al.* (2003) showed a significant increase of intracellular ROS by *in vitro* DCF treatment of hepatocytes, they observed such an effect at high concentrations of the drug (350 and 450 μmol/L) and the maximum effect was seen only after 5 h incubation. We measured the effect of DCF on ROS production at a lower and pharmacologically more relevant concentration. The divergent effect of DCF and rotenone on superoxide production may also underlie the differences that they exert on DA release.

Thus, while *in vitro* DCF mimics the effect of rotenone on striatal DA release (Milusheva *et al.* 2005), *in vivo* it acts substantially different. The ATP level was dramatically decreased in slices from chronically DCF i.v. treated animals compared with the sham-operated ones and the EC was lowered. Our data are in accordance with the finding of Bort *et al.* (1998) that DCF caused a fast depletion of ATP in hepatocytes. Krause *et al.* (2003) have also demonstrated uncoupling effect on mitochondrial respiration and inhibition of ATP turnover by DCF in intact cells. Similarly, inhibition of ATP biosynthesis by DCF was found in kidney mitochondria and cells (Ng *et al.* 2006).

However, despite the substantial energy depletion, the DA content was not significantly changed in *in vivo* DCF treated rats. The TH immunoreactivity showed high variance among individual rats, and a decrease was observed only in one third of the animals, whereas in others no change or increase was observed. The uptake of [^3^H]DA was enhanced, and DA release in response to H_2_O_2_ was diminished. HPLC analysis of release samples revealed significant changes in the effluent derived from the striata of DCF treated rats. However, these changes were different from those observed in our previous study using an identical protocol of *in vivo* rotenone treatment. In response to DCF treatment the proportion of DOPAC and DA decreased, whereas the proportion of TAs (OCT, TYR, and PEA) increased. TAs, are formed from l-phenylalanine and l-tyrosine by the aromatic amino acid decarboxylase and normally contribute to a minority of extracellular monoamine levels or remain undetectable (Branchek and Blackburn 2003; Lindemann and Hoener 2005). Therefore, increased formation of TAs together with the decrease in DA and DOPAC are indicative to a compromised synthesis of DA by the TH enzyme following chronic DCF treatment, which is accompanied by a shift of enzymatic activities to TA pathways. Although TAs also used to increase if their metabolism by monoamine oxidase enzyme were impeded, simultaneous DA accumulation in the effluent was not detected. It is also noteworthy that in response to DCF treatment we could not observe the appearance of DA quinone, the toxic oxidative metabolite of DA formed in the presence of H_2_O_2_ after *in vivo* rotenone treatment (Milusheva *et al.* 2005), and proposed to be a potential source of free radical-induced damage of dopaminergic terminals. However, our data raised the question whether the enhanced extracellular concentrations of TAs following chronic DCF treatment might be associated with a risk of neurological disorders for patients taking DCF over a prolonged period, as TAs dysregulation has been linked to various psychiatric diseases (Lindemann and Hoener 2005).

Taken together our *in vivo* data showed that chronic DCF treatment does not aggravate the effect of oxidative stress on dopaminergic transmission. It even decreased the susceptibility of striatal dopaminergic terminals to mild oxidative burden. In line with our *in vivo* data, we found marked difference in the intracellular superoxide generating ability of rotenone and DCF (see Fig 3b) and Sairam *et al.* (2003) have found reduction of hydroxyl radical generation by DCF. Recently, Dairam *et al.* (2006) have demonstrated the antioxidant and free radical scavenging properties of two other NSAIDs, tolmetin and sulindac, in hippocampal neurones. The authors have suggested that NSAIDs, by inhibiting cyclooxygenase activity, may act as indirect antioxidants, because cyclooxygenase activity itself produces ROS (Nikolic and Van Breemen 2001). This might be the case with our *in vivo* experiments. DCF was also shown to lower lipid peroxidation induced by Fe^2+^/ascorbate system and to accelerate decomposition of peroxynitrite (Mouithys-Mickalad *et al.* 2000).

A further possible explanation of the opposite *in vivo* and *in vitro* effects of DCF might be the actual concentration of the drug at the target site. As a low penetration through the blood–brain barrier was estimated for DCF (Dehouck *et al.*, 1992) it seems that *in vivo* DCF concentration in the striatum was much lower than the concentration we used *in vitro*. The variance in the intensity TH immunostaining might also reflect individual levels of metabolic activity resulting in a different rate of DCF decomposition *in vivo*. It was recently shown that brain penetration of NSAIDs, including DCF, might be modulated by cytokines (Fukuda *et al.* 2005) and therefore it may vary between individuals.

In conclusion, while *in vitro* DCF treatment mimics the effect of rotenone on striatal dopaminergic transmission, *in vivo* it rather counteracts than aggravates dopaminergic neurodegeneration. However, it should be kept in mind that it might be potentially toxic to individuals with altered integrity of blood–brain barrier which increases the risk of side-effects in the CNS.

## Abbreviations used

[^3^H]DA: [^3^H]dopamine
CM-H_2_DCFDA: 5-(and-6)-chloromethyl-2′,7′-dichlorodihydrofluorescein diacetate
DA: dopamine
DCF: diclofenac
DMSO: dimethylsulfoxide
DOPAC: 3,4-dihydroxyphenylacetic acid
EC: energy charge
HEt: hydroethidine
HVA: homovanilic acid
NSAID: non-steroidal anti-inflammatory drug
OCT: octopamine
PEA: β-phenylethylamine
PEG: polyethylene glycol
ROS: reactive oxygen species
TA: trace amine
TH: tyrosine hydroxylase
TYR: tyramine
